# Defects in graphene-based heterostructures: topological and geometrical effects

**DOI:** 10.1039/d1ra08884j

**Published:** 2022-02-28

**Authors:** Lei Fan, Jin Xu, Yihong Hong

**Affiliations:** School of Civil Engineering and Architecture, Zhejiang University of Science & Technology Hangzhou PR China fanleigl@foxmail.com xujin@zust.edu.cn; Shanghai Urban Construction Vocational College Shanghai China hyhzju@126.com

## Abstract

The combination of graphene (Gr) and graphene-like materials provides the possibility of using two-dimensional (2D) atomic layer building blocks to create unprecedented architectures. The most attractive characteristics are strongly dependent on the various spatial structures, mainly including in-plane heterostructures butt-joined at the side of an atomic monolayer through covalent bonds, van der Waals (vdW) heterostructures involving a vertically stacked hybrid structure, and their combinations. Heterostructures can not only overcome the limitations inherent to each material but may also obtain new features by appropriate material combination. However, heterostructures made of vdW force superposition or covalent bond splicing are prone to defects. The introduction of external and internal defects causes local deformation and stress in the material, thereby affecting the physical properties of the material, such as its transport properties and mechanical properties. Therefore, research, utilization and control of these defects are highly critical. This paper reviews the vacancy, topological and geometrical effects of defects in modulating the structures and mechanical responses of Gr-based heterostructures. Moreover, the coupling effects of various defects on the Gr-based heterostructures in multi-physics fields are also discussed. This work aims to improve the understanding of the physical mechanism of defective configurations and their association in low dimensions, so as to realize various configurations and to aid the search for new usages.

## Abstract 2

The combination of graphene (Gr) and graphene-like materials provides the possibility of using two-dimensional (2D) atomic layer building blocks to create unprecedented architectures.

## Graphical abstract

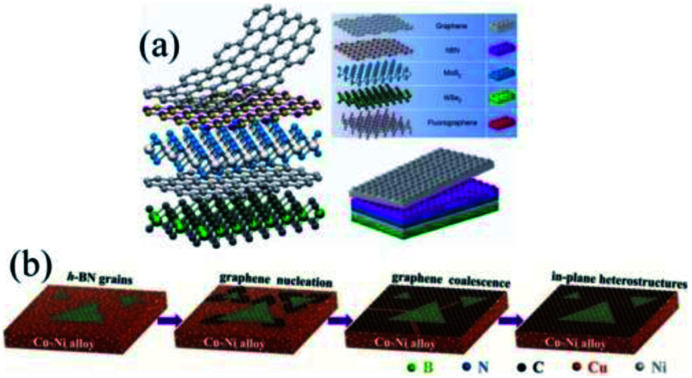

## 1 Introduction

**Fig. 1:**
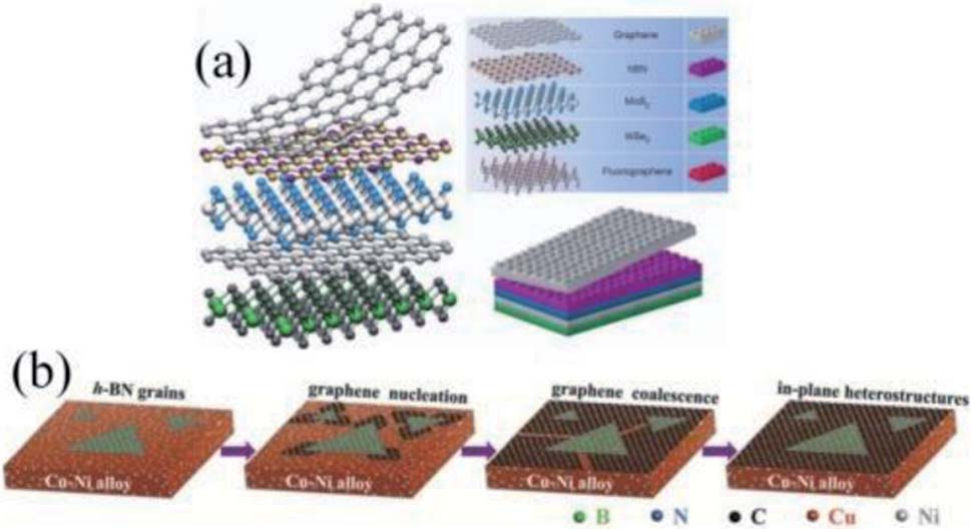
Spatial configuration of a heterostructure. (a) Schematic of a vdW heterostructure.^8^ (b) Schematic of an in-plane heterostructure.^11^

2D materials, due to their eminent electrical,^1,2^ optical,^3,4^ and mechanical properties,^5,6^ have aroused great interest. They benefit from weak interlayer van der Waals (vdW) interactions^7–9^ and strong in-plane covalent bonds,^10–12^ as shown in Fig. 1. Multiple 2D materials of the same or different dimensions can be stacked in a specific way (like Lego bricks) or spliced together.^13,14^

Most graphene/h-BN heterostructures (Gr/h-BN) are limited to a single heterostructure or the inner surface of a vertical structure, regardless of the combinations thereof, which can produce interesting heat-transfer characteristics that depend on the design having a high density of heterogeneous multi-pixel functions.

Hexagonal boron nitride (h-BN) and Gr have a highly homologous configuration but different electrical properties. The integration of Gr and Gr-like materials can create fascinating heterostructures. Moreover, such heterostructures can not only overcome the intrinsic restrictions of lone materials but may also lead to new performance being acquired by suitable combinations thereof.

h-BN is a dielectric material (band gap = 5.9 eV) but Gr has no band gap and is considered to be a semiconductor.^15,16^ The band gap of Gr can be opened by the hybridization of the two materials. In addition, vdW heterojunctions provide two additional adjustable degrees of freedom, namely, the number of layers and the angle between layers.^17^ By adjusting these two additional degrees of freedom, the energy band structure and electronic transport characteristics of the system can be changed.^18^ In 2014, Woods *et al.* found that the moiré fringes are formed in the Gr/h-BN hybrid structure by rotating it at an angle. Meanwhile, a commensurate–incommensurate transition for a hybrid structure has been realized.^19^ In March 2018, Nature magazine published two findings on Gr superconductivity: two new electronic states were found in the twisted stacking double-layer Gr heterojunction, which could easily realize the transition from an insulator to superconductor.^20,21^ In February 2019, Nature magazine published three articles, which reported the first independent, experimental observation of the existence of a moiré exciton state in 2D heterostructures by several research groups in the United States.^22–24^ In 2020, by controlling the twist angle, the “magic angle” characteristics of the Gr-based materials could be extended to other 2D research systems, and the distribution information on the twist angle was also studied.^25,26^ Based on a series of discoveries about the “magic angle”, it could be expected to realize the performance of a “superconductor” at room temperature, and this set off a huge wave of interest in such fields as energy, electronics, environmental science, and computer industry. However, when a defect and torsion angle coexist, what effects do they have on the interfacial structure, and phonon and electron transport of a vdW heterojunction? The interaction, load transfer, and structural stability of vdW heterostructures with different spatial structures are affected by stress and local deformation caused by various defects. Due to the difference in stacking mode and twist angle, a coupling effect (defect and physical field) will occur, which will expand or reduce the performance of vdW heterostructures.

It is easy to form various kinds of defects in the process of nucleation and growth, which can then form defect clusters in the process of use, which could ultimately change the physical properties of 2D crystals. Compared with three-dimensional (3D) bulk materials, the physical properties of 2D materials, especially their transport behavior and mechanical properties, are more susceptible to local deformation and stress caused by internal and external defects.

Therefore, how to recognize the characteristics of these defects and how to use and control them are very important. This paper reviews the vacancy, topological, and geometrical effects of defects in modulating the structures and mechanical responses of Gr-based heterostructures. Moreover, the coupling effect of heterojunction defects in the multi-physics field is also discussed in this paper.

## 2 Effects of defects in graphene-based heterostructures

The structural defects of Gr-based materials can be divided into two categories: intrinsic defects, which are composed of non-sp^2^ hybrid carbon atoms in the 2D structure,^27^ and introduced defects, which are composed of other atoms, such as boron (B), nitrogen (N), or oxygen (O), in the form of covalent bonds between carbon (C) atoms and the 2D structure.^28^ In addition, graphene vertically stacked heterostructures will form defects when multiple 2D materials are vertically stacked through the weak interactions of the vdW force. Also, graphene planar heterostructures will also form defects when two different atomic monolayers are seamlessly stitched together through covalent bonds. For example, these various defects will affect the electrical, optical, and mechanical properties of Gr-based heterostructures.^29^ The appearance of defects changes the bond length and types of some atomic hybrid orbitals. Changes in the bond length and orbit can change the electrical characteristics of the defect region. Point defects and single-vacancy defects form electron wave scattering centers on the surface, which affect the transmission of electrons and eventually reduce the conductivity. In addition, changes in the bond length and bond angle will affect the anharmonic interactions of atoms in the composite system, and then affect the phonon group velocity and phonon-transport efficiency.^30^ Defects will also produce stress accumulation and promote the out-of-plane displacement, thermal fluctuation, and local bending of Gr-based materials.^31^ As a result, the deformation of the structure of Gr-based materials will affect the thermal and electrical transport properties.

### 2.1 Vacancy effects of defects in graphene-based heterostructures

Chemical vapor deposition is considered to be the most effective and high-quality technology for the preparation of graphene and graphene heterojunctions, but chemical vapor deposition still leads to inevitable inherent structural defects with different types and sizes. Vacancy defects may occur in the process of chemical vapor deposition. In addition, the process of stripping graphene or a graphene heterojunction from the metal surface is also prone to causing vacancy defects. By using ion irradiation, the defects of graphene can be effectively controlled to obtain the desired properties.^32^ When an electron beam with appropriate energy bombards the surface of graphene, the carbon atoms on the graphene will break away from the system constraints due to the action of energy.^33^ These carbon atoms will completely leave the surface of graphene, and then form vacancy defects.^33^ Ion irradiation is also suitable for regulating the defects of Gr-based materials so that the required properties can be obtained.^34,35^

**Fig. 2:**
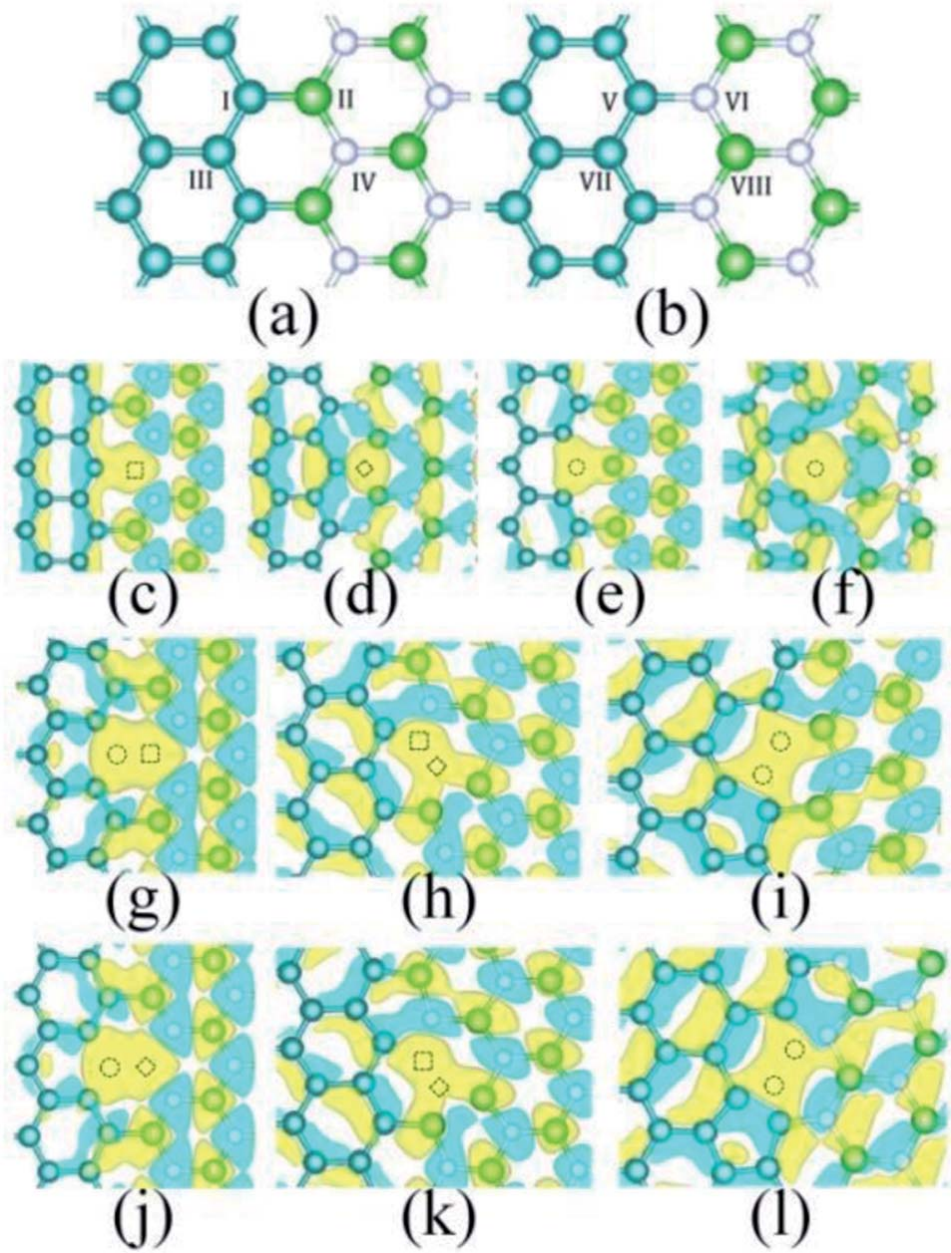
Spatial structures of zigzag interfaces with (a) defective-carbon–boron bonds and (b) defective-carbon–nitrogen bonds. The ground state configuration and charge-transfer profile of single-vacancy: (c) defective boron, (d) defective nitrogen, (e) defective carbon neighboring boron, (f) single-vacancy-carbon neighboring nitrogen in a zigzag interface. The ground state configuration and charge-transfer profile of double-vacancy: (g) defective carbon–boron, (h) defective boron–nitrogen and (i) defective carbon–carbon at the defective Gr/h-BN interface with C–B bonds in the zigzag direction, (j) double vacancy carbon–nitrogen, (k) double vacancy boron–nitrogen and (l) double vacancy carbon–carbon at the defective Gr/h-BN interface containing defective-carbon–nitrogen bonds in the zigzag direction.^37^

Vacancy defects can be divided into single-nanohole, double-nanoholes, and multiple-nanoholes. In 2018, M. Y. Li^36^ investigated the influence of single-void defects on the heat conduction of the Gr/h-BN interface. Their findings showed that the change in vacancy concentration had a direct relationship with the interfacial thermal conductivity. Using stress analysis and power spectrum analysis, the deterioration of the in-plane mode was shown to be caused by a single vacancy.^36^ Obviously, the loss of an atom will inevitably cause the rupture of the three covalent bonds connected to it. As a result, three dangling bonds are formed. Under the influence of the Jahn–Teller effect, the atoms lost at the interface of a heterojunction will undergo structural re-arrangement and energy variation. O. Y. Bin^37^ showed that the formation energies of the three single-vacancy defects defective-carbon, defective-boron, and defective-nitrogen atoms in the zigzag direction were 6.08, 8.29, and 7.23 eV, respectively. The formation energies of the double-vacancy defects defective-carbon–carbon, defective-carbon–boron, defective-carbon–nitrogen, and defective-nitrogen–boron bonds in the zigzag direction were 5.35, 10.25, 8.41, and 10.17 eV, respectively. The formation energies *E*_tot_ of SV_*α*_ and DV_*βγ*_ are assumed to be as follows:^37^1
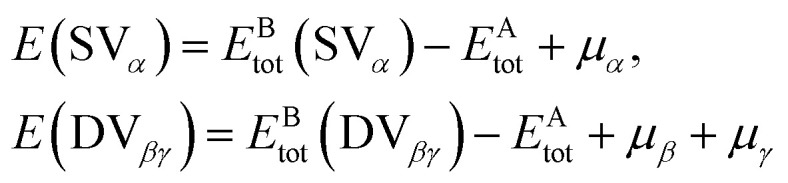
where *E*^A^_tot_ is the system energies with free-defects, and *E*^B^_tot_ is the system energies with vacancy defects. When the corresponding atoms i (C, B, or N) are eliminated, their chemical potential can be represented by *μ*_i=*α*,*β*,*γ*_.^37^Fig. 2 shows the spatial structure of atoms and the charge profile of the defective Gr/h-BN interface.

For vertical heterostructure, due to the diversity of its spatial structure, this defect can be regarded as a control method for planning the next generation of high-performance electronic equipment and vdW heterostructure-enabled nanosensors. M. L. Sun *et al.*^38^ employed first-principles calculations to study the spatial configuration and electrical transmission performance of a WSe_2_/Gr hybrid structure with vacancy nanoholes. Their findings indicated that the performance of a WSe_2_/Gr hybrid structure was greatly transformed when vacancy defects were introduced. A better device property can be acquired by drawing the vacancy defects in the Gr/WSe_2_ interface, and then reducing the potential barrier of the interface.^38^ In another work, C. J. Alvarez *et al.* used the direct growth of both monolayers method to prepare a MoSe_2_/Gr vdW heterostructure.^39^ Moreover, the evolution of the growth processes and their effects on the formation and movement of external defects were studied.^39^ Additionally, in 2018, S. Y. Kim *et al.*^40^ employed experimental and computational investigations to explore the effect of substrate surface defects on the properties of 2D vdW structures. Their findings indicate that the imperfection of Gr have no crucial effect on the configuration or mass of the grown WS_2_ sheet, but it has the crucial effect on the mutual effect between stacked stratum, thereby affecting the overall properties of the layers.^40^ In 2021, W. Li *et al.*^41^ investigated the electronic and energy performance of imperfections in a Gr/MoS_2_ vdW heterojunction by DFT theory. The results show that the energy of carbon imperfection was decreased by 0.06–0.54 eV, while that of S imperfection increased by 0.36–0.53 eV.^41^ It is worth noting that the influence modes of imperfection on planar heterojunctions and vdW hybrid nanostructures were different; for instance, imperfection in the most momentous section of the planar h-BN/Gr hybrid nanostructure of two monolayers could influence the mechanical performance. The mechanics performance of planar h-BN/Gr nanostructures is affected by the connection method and chemical bond strength. However, vdW heterojunctions are affected not only by chemical bonds, but also by the vdW force. Therefore, under the action of external force and defects, the load transfer and fracture modes of vdW and planar heterojunctions are different.

### 2.2 Geometrical effect of defects in graphene-based heterostructures

**Fig. 3:**
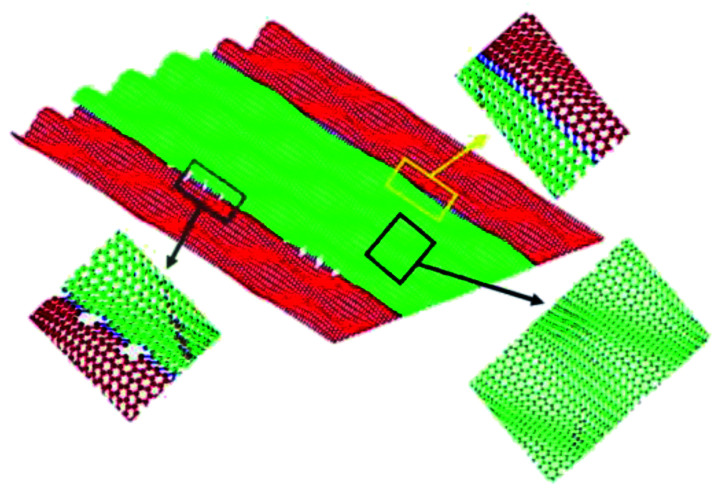
Two types of geometric imperfection were established in the boundary of the Gr–BN–Gr planar heterostructures.^42^

When graphene-based heterostructures are deposited and grown on the surface, it usually nucleates in many places at first. Then, the crystals grow separately until they meet. Because the interaction between graphene-based heterostructures and most substrates is weak, and the substrate surface usually has a polycrystalline texture, it is difficult to have the same crystal orientation between the growth nuclei and their crystal domains. Next, when growing single-crystal nuclei that meet at the boundary and form continuous films, topological defects are easy to form in this process. The geometric effects of topological defects on Gr-based heterostructures are shown in two aspects: from the perspective of the geometric shape, and the effects that defects with different geometric shapes have on the properties of Gr-based heterostructures. In 2018, E. E. Kasra demonstrated that two kinds of geometric defects (circular and square nanoholes) are created in Gr–BN–Gr planar heterostructures,^42^ as shown in Fig. 3. Their research showed that circular nanoholes have less effect on the mechanical properties of Gr–BN–Gr planar heterostructures, compared with square imperfections. The weaker mechanical properties in the presence of square nanoholes was also attributed to the higher stress concentration and removal of more atoms.^42^ In addition, the orientation of geometric defects (longitudinal and transverse) has different influences on the mechanics performance of Gr/h-BN hybrid nanostructure. This can be due to the different orientation and stretching direction of the imperfections in hybrid nanostructures.^43^ In 2019, L. Fan used molecular dynamics simulation to explore the effect of interlayer sp^3^ bonds and geometric defects on the mechanical properties of h-BN/Gr stacked hybrid nanostructures. In this work, the negative effect of rhomboid defects on the mechanical properties of the heterostructure was weaker than that of square defects.^44^

The existence of geometric imperfection is helpful to produce many novel physical phenomena and properties in Gr-based heterostructure. In 2020, the influences of geometry on the thermal resistance of h-BN/Gr planar hybrid nanostructures were investigated by MD simulation.^45^ The results showed the addition of interface thermal resistance led to either excessive phonon dispersion, or a decrease in phonon diversion, which was caused by the existence of geometric imperfections in the Gr/h-BN planar heterostructures.^45^ Besides, the geometric effect of the defect was also closely related to the position of the defect (bonds energies). In another work, a series of geometric imperfections were established in diverse locations of h-BN/Gr planar heterostructures, including h-BN (B–N bonds), Gr (C–C bonds), and the interface (B–C bonds).^46^ The results showed that it was better to place these circular nanopores in the Gr domain rather than the interface and h-BN domain in order to obtain better ductility and strength.^46^ A key conclusion was drawn that the bonding energy of C–C was 607 kcal mol^−1^, while the bonding energies of B–N and C–B were 389 kcal mol^−1^ and 448 kcal mol^−1^, respectively.^47^

Gr-based heterostructures not only depend on the geometric size, defect location, and interface connection, but also the defect structure. The geometric effects of defects, which induce internal stresses in materials, can also lead to geometric deformation effects on structures. Z. G. Song and Z. P. Xu^48^ investigated the defect-induced geometric effect of Gr; whereby changes in the local bending structure (the shape of the cone and saddle with a positive and negative curvature) of Gr changed its equivalent tensile and bending stiffness.^48^ In another work, by constructing a polycrystalline Gr model with various topological defects at the grain boundaries (GBs), the relationship between out-of-plane displacement and local mechanical response is discussed. Defects with a positive Gauss curvature had the effect of stiffening and strengthening under indentation tests, while defects with a negative Gauss curvature softened and weakened.^49^ Like with Gr, such a geometric effect of Gr-based heterostructures may be induced by defects. The local out-of-plane deformation (caused by these defects) has a critical influence on the local mechanics response of Gr-based heterostructures.

### 2.3 Topological effect of defects in Gr-based heterostructures

**Fig. 4:**
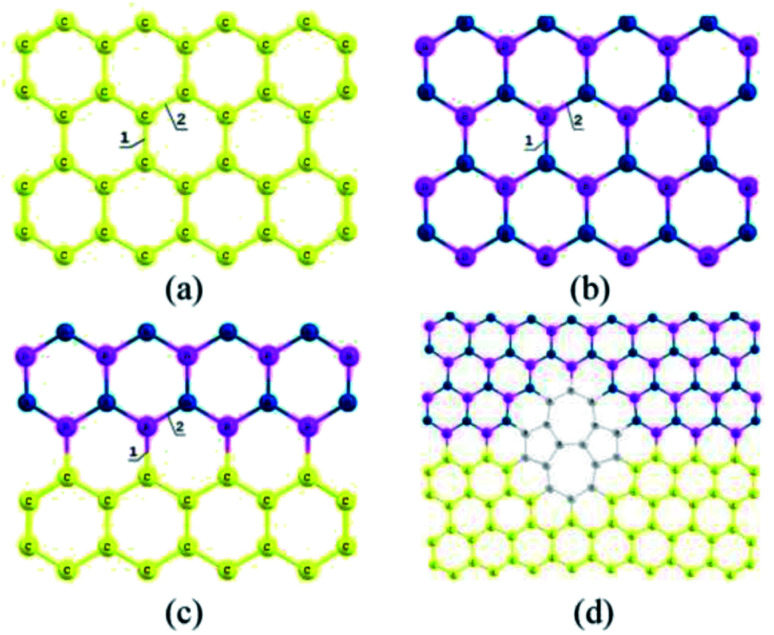
Geometric structures of 2D materials: (a) Gr sheet, (b) h-BN sheet, (c) defect-free Gr/h-BN planar heterostructures and (d) Gr/h-BN planar heterostructures with Stone–Wales defects.^53^

The Stone–Wales (SW) defect is one of the most common topological defects in most planar low-dimensional materials, in which the defect structure retains the number of atoms in the base system without forming any dangling bonds.^50^ The original regular hexagonal network structure will change to an irregular non-hexagonal structure by introducing topology defects.^51–53^ The establishment of SW defects depends on the rotation of carbon–carbon or boron–nitrogen bonds,^53^ as shown in Fig. 4.

The formation energies, and bond angle and dimensions will be changed by introducing the SW imperfection in the Gr/h-BN planar hybrid nanostructure.^53^ For example, the carbon–carbon bonding of defect-free Gr nanostructures (1.44, 1.43 Å) are equal or smaller than those of pristine Gr/h-BN planar hybrid nanostructures (1.45, 1.43 Å). The carbon–carbon bonding at the SW imperfection (1.47, 1.44 Å) of Gr/h-BN planar hybrid nanostructures is bigger than in the case of defective Gr (1.36 Å). In addition, the formation energies of the Gr/h-BN planar heterostructures in the presence of SW defects were 4.05 eV, and the range of formation energy followed a certain rule: Gr < Gr/h-BN planar heterostructures < h-BN.^53^ Defect formation energies (*E*) can be expressed by the following formula:

2 *E* = *E*_SW_ − *E*_pristine_

where *E*_pristine_ is the total energy of the pristine nanostructure and *E*_SW_ is the total energy of the defective nanostructure in the presence of SW imperfection.

SW defects can be regarded as a dislocation dipole. The initial stress field produced by the dislocation dipole determines the strength of the GBs. GBs are always inevitable.^54–58^ When two materials with different orientations are spliced together, GBs at the interface represent the weak link of the material. These defects will determine the performance of the material. Therefore, it is crucial to know what topological effects the imperfection in defective Gr and other 2D materials will have on their thermal, mechanical, and electrical properties, and what mechanical loads and pressures the defective hybrid materials made of these two 2D materials can bear.

Liu *et al.* found that the influence of topological defects on Gr and Gr-based heterostructures (Gr/h-BN) is different.^59^ The thermal conductivity of polycrystalline Gr will be reduced due to the phonon backscattering caused by atomic defects,^60^ while the thermal conductance of h-BN/Gr hybrid nanostructure will be abnormally enhanced by introducing topological defects at the interface of these two materials. This abnormal enhancement of interfacial thermal conductivity can be attributed to the fact that the stress field is localized due to a mismatch dislocation at the interface and its out-of-plane deformation.^59^ In another work, the thermal transfer efficiency of GBs at the interface of Gr/h-BN heterostructures was reported to not only be related to the direction of heat flow, but also related to the grain misalignment angle. A key conclusion was found that the heat-transfer efficiency from h-BN to Gr is lower than that from Gr to h-BN.^61^ In addition, the arrangement of 5–7 disclinations along the GB is very important for the mechanical strength, and the stress concentration at the GB decreases with the increase in disclination density.^61^ R. Daniel demonstrated^62^ that both external and internal disorders are related for vdW hybrid nanostructures composed of Gr and transition metal dichalcogenides. In 2017, P. R. Bandaru^63^ investigated the characteristics and topology of defects on 2D materials surfaces and their influence on charge and energy storage. Their research indicated that topological defects can induce different bond orderings and bond configurations, which then affect the capacitance and the magnitude of the energy storage. The in-plane sp^2^ bonding on each constituent graphene sheet of the graphite involves pendant π-groups, which may be indirectly involved in charge transfer through weak bonding and adsorption processes.^64^ Furthermore, the effect of topological defects on the electronic characteristic of planar CBN heterostructure was investigated by S. Thomas *et al.*^65^ The results showed that CBN heterostructures with original defects and SW defects retained the electronic characteristics of direct semiconductors. Interestingly, the single vacancy of C and B makes them metals, which is due to the slight overlapping of the bands at the Fermi levels.^65^

The short linear stacking of topological defects or other types of defects, such as nanoholes or chemical modifications,^48,66^ will generate stresses that increase with the length of the arrangement within the Gr-based heterostructure. Because of the complex mechanism of failure behavior and a great many influencing factors, including the energy at the GB, initial stress (caused by SW defects), stress gradient and bending angle (caused by the GB), these factors have a certain regulatory function on the properties of the 2D material, and are different from the behavior in bulk materials. Therefore, how to accurately predict and control the effects of the failure behavior and thermal properties of defective Gr-based heterostructures is a pressing problem which needs to be solved.

### 2.4 Quasi-3D effect of defects in graphene-based heterostructures

**Fig. 5:**
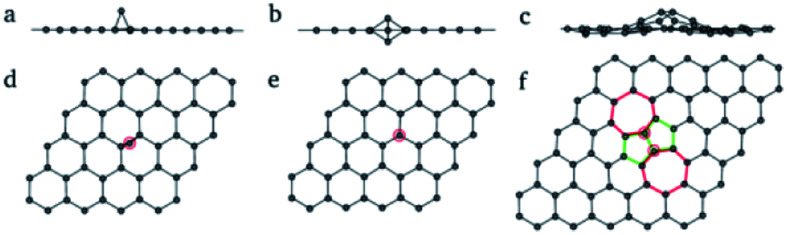
Carbon atoms defects in Gr: (a–c) space configuration; (d–f) positions of carbon defects.^70^

Carbon atoms lost during the formation of single-holes and multiple-holes are not necessarily completely separated from Gr.^67^ The carbon atoms may form delocalized atoms, and then migrate on the Gr surface after leaving the original 6-membered ring.^68^ These defects (delocalized atoms) will change the type of orbital hybridization of carbon atoms, in turn resulting in the appearance of sp^3^ hybrid orbital carbon in Gr.^69^ The introduction of defects in out-of-plane Gr is shown in Fig. 5.

Under the condition of chemical vapor deposition or strong oxidation, the introduction of metal atoms^71,72^ or oxygen-containing functional groups^73,74^ on the surfaces of Gr is inevitable due to the use of metal elements or oxygen-containing oxidants. These heteroatoms are connected with carbon atoms in Gr by strong chemical bonda or weak vdW force, forming out-of-plane heteroatom defects.^27^

**Fig. 6:**
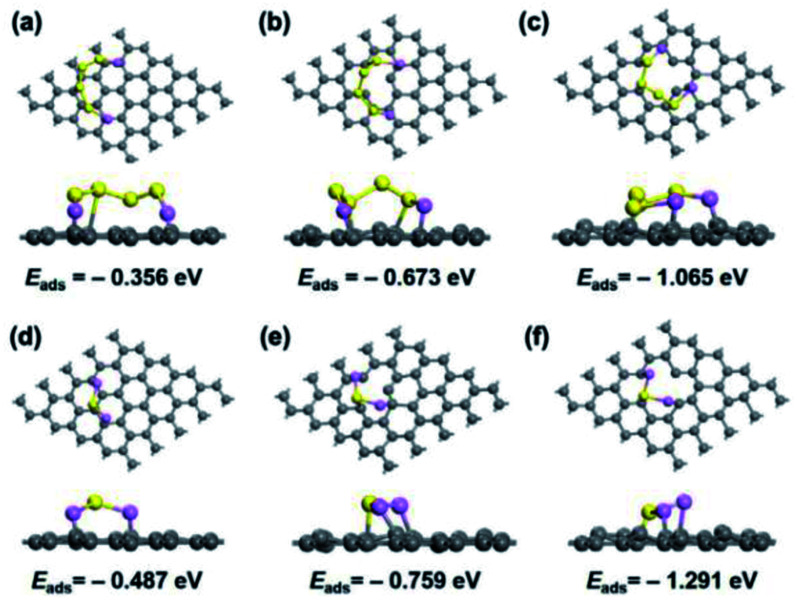
Binding configurations and related adsorption energies of (a–c) Li_2_S_4_ and (d–f) Li_2_S on pristine graphene ((a) and (d)), monovacancy graphene ((b) and (e)), and divacancy graphene ((c) and (f)).^80^

Based on the preceding research, Zhang Y. Y. *et al.*^75^ calculated the influence of sp^3^ bonding on the physical properties of the bilayer Gr nanostructure. Their findings showed that the sp^3^ bonding has two opposite effects on the physical properties of the bilayer Gr nanostructure. On the one hand, the sp^3^ bonding can stimulate a reinforcement effect for the load transfer ability and shear modulus of the bilayer Gr sheets. On the other hand, the tensile stress of the bilayer Gr nanostructure will be reduced due to the local strain induced by sp^3^ bonding.^75^ In addition, the thermal transport of bilayer and multilayer Gr nanostructures can be controlled by introducing sp^3^ bonding.^76^ In 2015, Y. F. Gao calculated the influence of sp^3^ bonding on the thermal transport properties of a Gr-like structure (bilayer boron nitride).^77^ They also that the showed thermal transport properties of bilayered Gr could be effectively controlled by modifying the interlayer bonding. T. Iwata^78^ indicated the possibility of tuning the thermal conductivity values of bi-Gr and bi-BN by establishing sp^3^ bonding. Many researchers then began to pay attention to the interlayer bond, especially whether it can bring more novel physical phenomena to the Gr hybrid nanostructure. By studying the in-plane and out-of-plane geometric deformations caused by interlayer bonding, the mechanical model of for a Gr optimal design with the target spatial structure was established by Fan *et al.* They expected that the interlayer bonds of Gr-based heterobilayers can play a role in defects for Gr and 2D materials.^79^ In 2020, W. L. Cai *et al.*^80^ used experiments and density functional theory to study the effect of defect engineering on carbon black for accelerated Li–S chemistry. In this work, the spatial configurations, bonds structure, and related adsorption energies could be effectively regulated by using defect engineering (see Fig. 6). In another work, the electrocatalytic activity of the carbon architecture in Li–S chemistry was manipulated by using a supercritical CO_2_ foaming technique and defect engineering.^81^ The findings in that study are of great significance for understanding and deciphering the electrocatalytic mechanism of the carbon architecture for the development of advanced Li–S battery applications.^81^

## 3 Coupling effect of defects in multiple physical fields

As mentioned above, defects will produce many novel physical effects, including vacancy, quasi-3D, topological, and geometrical effects and so on. If there are two or more defects in nanostructures at the same time, what coupling effect will the internal field involving the vacancy defect, SW defect, special defect (interlayer sp^3^ bonds, hydrogenation, functional group and so on) have on the physical properties of Gr-based materials? In addition, the effects of the external field^82^ (temperature, strain, electric field, *etc.*) and internal field on the physical properties of Gr-based heterostructures are also important, because the practical application of Gr-based heterostructures involves the interaction of multiple physical fields, such as heat transfer, vibration, airflow, and compression. In 2014, N. N. Li^83^ used molecular dynamics simulations to investigate the effects of hydrogenation and the grain boundary on the mechanical properties of polycrystalline Gr. The results revealed that the failure stress of polycrystalline Gr with a hydrogenated atom was remarkably decreased by the associated shortening effect of bond pre-straining in sp^3^ atoms and the multiple defective GBs.^83^ In 2017, E. B. V. Jose^84^ employed first-principles calculations to study the effects of hydrogenation and the grain boundary on the charge transport of hydrogenated polycrystalline graphene, and found that the charge transfer was weakly sensitive to hydrogenation when the adsorbate was confined to the grain boundary. However, the uniform distribution of hydrogen reduced the electron mobility. This difference was due to the formation of hydrogen-induced resonant impurity states.^84^ Therefore, nanoholes, defects, and chemical modification will produce a local stress field, and its amplitude has a certain random distribution in actual material samples. Also, a tunability of the electrical transport of Gr-based materials can be realized through a selective “defect” coupling effect. These studies and related discoveries raised a series of questions for material physics research, and also provided opportunities for the preparation and design of micro–nano materials and structures. In 2018, A. R. Wei^85^ used a reverse nonequilibrium molecular dynamics method to study the effect of the grain boundary and hydrogenation on the strain-dependent thermal conductivity of hydrogenated polycrystalline graphene under the condition of a strain field. The results showed that the thermal properties of hydrogenated polycrystalline graphene under tension load were related to the average stress in the hydrogenated polycrystalline graphene due to the softening of the phonon modes.^85^ This could be attributed to the fact that both hydrogenation and oxidation will gradually change the material from a honeycomb to a diamond-like structure as result of the hybridization transition from sp^2^ to sp^3^, while the introduction of grain boundary will only lead to the distortion of sp^2^ hybridization.^86^ Also in 2018, using molecular dynamics, the coupling effects of an external field (temperature and strain) and internal field (longitudinal and transverse defects) on the mechanical properties of Gr/h-BN–Cu heterostructures were investigated by L. Fan *et al.*^87^ As a result, atoms near the defect are more likely to escape covalent bond forces when the temperature increases. Moreover, these “defective atoms” are in a semi-active state, and the atoms seem to be applied with an “escape force”.^87^ The geometric morphology of the Gr-based heterostructure depends not only on the thermal disturbance of the environment, but also on the defect structure.

Similarly, in 2021, L. Fan *et al.* used molecular dynamics to study the effect of special defect (interlayer sp^3^ bonds), intrinsic defects, and the stacking angle on the interfacial thermal transport of Gr/h-BN heterostructures.^88^ Their findings indicated that vdW heterostructures are gradually transformed into quasi-3D nanostructures by introducing interlayer bonding. Furthermore, the interlayer sp^3^ bond acts as a special defect, and the defect and interlayer bond form a defect amplification effect. Also, in 2020, W. J. Yao *et al.*^89^ employed molecular dynamics to study the effects of an external (temperature and tension load) and internal (interlayer bonds and nanopores) field on the mechanical properties of Gr/h-BN heterostructures, and found that the atoms close to interlayer bonds and nanopores were more likely to get out of the equilibrium position due to the built-in distortion stress field generated by the coupling effect of temperature and tension loading.^89^ In another work, the mechanical properties of Gr/h-BN heterostructures with interlayer bonds and SW defects under the condition of high temperature and load were investigated.^90^ The study findings indicated that the negative effect of interlayer bonding and SW defects on the mechanical properties of the quasi-3D spatial configuration could be accelerated by manipulating multiple physical fields (tensile strain and temperature).

Hence, by studying the response and stability of defective 2D materials under an external field (temperature and load), a physical model for the optimal design of the spatial structure can be established. The quasi-3D effect induced by interlayer bonding and the in-plane stress and structural deformation induced by topological defect may produce more attractive physical effects. These physical effects are particularly significant in high temperature and loading environments, and are different from the behavior in 3D bulk materials, so the physical mechanism is worth exploring.

## 4 Conclusion and future prospects

In practical engineering, Gr-based heterogeneous materials may experience the superposition of physical fields (electrical–thermal, thermal–mechanical, and acoustic–structural coupling, *etc.*), and these physical fields will interact with each other. Gr-based heterogeneous materials inevitably contain various defects, and these defects will affect the properties of Gr-based heterostructures. In the case of multi-field coupling, what effect will the defects have on Gr-based heterostructures?

### 4.1 Defect control

Because the inherent symmetry of the Gr honeycomb lattice is destroyed, point defects induces local states near the Fermi level, which leads to energy splitting at the Dirac point. As a result, the conductivity of Gr gradually decreases, and carrier resonance scattering also occurs.^91^ However, the defects and nanoholes are not all harmful.^92^ The defects improve the semiconductor performance, ion-diffusion coefficient, dispersivity, and reaction activity of Gr-based materials. These defects can be controlled actively, which can then give full play to the advantages of defects in Gr-based materials. Particle irradiation technology can modify and cut Gr-based materials, and change their physical and chemical properties according to the engineering needs. In the process of irradiation, Gr-based materials can be damaged by changing the dose, type, energy, and incident angle of the incident particles, thus affecting the performance of Gr-based materials.^93–95^ Particle irradiation (induced defect generation) has aroused great concern in the field of 2D materials.^96^ Hence, it is important to understand the issues and research, including (a) the interaction mechanism between energetic particles and micro–nano targets, (b) the formation theory of structure evolution in Gr-based materials induced by a particle beam, (c) lattice-oriented evolution of the hybrid structure, and (d) the fine control principles of the hybrid structure.

### 4.2 Defect coupling and active amplification mechanism

Many kinds of defects (zero-dimensional defects, one-dimensional defects, 2D defects, and introduced defects, *etc.*) may be formed in the use and preparation of Gr-based materials. It is well known that one type of defect will affect the performance of the system. If there are two or many kinds of defects at the same time, what will happen to the performance of the system? Gr-based materials are more likely to exceed the bound energy limit, and hence can get out of the stable state under the condition of an external field (temperature, strain, and so on). Defects coupling may also produce an active amplification mechanism of the defects due to local deformation and stress (induced by nanoholes or defects).

### 4.3 Interlayer defects and structural design

The diverse interface structures of Gr-based heterostructures, typically including vdW hybrid nanostructures that involve stacking Gr-based nanostructure like building blocks through vdW force, create the opportunity for diverse and tunable properties. For example, the spatial configuration of Gr-based materials can be optimized by using hydrogen bonds, ion bonds, and polymer cross-linking. These defects not only change the geometric structure (out-of-plane and in-plane geometric deformation) of Gr-like materials, but also affect the load transfer and transport process. In the preparation of vdW heterostructures, the combination of layers often has a certain design of significance for constructing macroscopic materials. It provides a variety of spatial configurations, and even allows exploring new uses through the regulation of defects.

## Abbreviations

vdW: van der Waals
2D: Two-dimensional
3D: Three-dimensional
Gr: Graphene
h-BN: Hexagonal boron nitride
Gr/h-BN: Graphene/h-BN heterostructures
GBs: Grain boundaries
SW: Stone–Wales
B: Boron
N: Nitrogen
O: Oxygen
C: Carbon

## Conflicts of interest

No potential conflict of interest was reported by the authors.

## Supplementary Material
